# Training of Faculty and Staff in Recognising Undergraduate Medical Students’ Psychological Symptoms and Providing Support: A Narrative Literature Review

**DOI:** 10.3390/bs12090305

**Published:** 2022-08-25

**Authors:** Costas S. Constantinou, Tinna Osk Thrastardottir, Hamreet Kaur Baidwan, Mohlaka Strong Makenete, Alexia Papageorgiou, Stelios Georgiades

**Affiliations:** Department of Basic and Clinical Sciences, University of Nicosia Medical School, Nicosia 2414, Cyprus

**Keywords:** mental illness, medical students, higher education, training, faculty/staff

## Abstract

Mental illness among medical students in particular, and higher education students in general, is very high. Many measures have been suggested in order to improve the situation, including training members of faculty and staff. However, to the best of our knowledge there seem to be no studies proposing training programmes for medical schools’ faculty and staff in response to recognising students’ psychological difficulties and symptoms in order to provide the relevant support. Also, in cases where such training for supporting medical students with psychological symptomatology exist, the efficacy of the approach employed is not known. By employing a careful literature search according to published guidelines for narrative literature reviews, this study aimed to fill in this identified gap in the literature. From the literature search, 14 articles were included in this review and the results show that no training tailored for faculty and staff in medical schools was identified. However, articles that related to higher education were included because they were useful in providing insights for medicine, and show the following: (a) faculty and staff acknowledged the importance of mental illness among students, (b) many of them discussed with their students psychological symptoms and provided support, (c) they tended to feel unprepared for recognising students’ psychological symptoms successfully and providing support, (d) they embraced the idea of being trained, and (e) any training seemed to be helpful for members of faculty and staff. From the results of this narrative review, we propose the CReATE circular pathway to ensure a sustainable process of training and support for students’ development.

## 1. Introduction

The British Medical Association (BMA) [1] published a press release in 2018 entitled “Medical students must be given better mental health support to prepare them for emotional toll of career in the NHS”. The press release highlighted the high rates of mental illness among medical students and based on the “shocking statistics” of this issue called for the need to enhance students’ psychological support [1]. The BMA stressed out a problem which has been well documented in the literature. More specifically, Rotenstein et al.’s [2] systematic review and meta-analysis of mental illness among medical students explored the findings of 167 studies with 116,028 participants from 47 countries. The review showed that 27.2% of students had depression, while 11.1% of them had suicidal ideation. Such findings were alarming because they indicated the risk of suicide and highlighted the need for further inspection. Along similar lines, 10% of medical students in Karp and Levine’s study [3] experienced burnout and suicidal thoughts. A survey by Student BMJ revealed that 30% of medical students reported mental illness and 15% had suicidal ideation [4]. More recent evidence provides similar results. Azim [5] explained that research on mental illness among medical students has shown relatively consistent results over the years. Zeng et al.’s [6] meta-analysis of ten studies in China with a total of 30,817 participants revealed that mental illness was very high among medical students. Prevalence of depression was 29%, anxiety 21%, and suicidal ideation 11%. Maser et al. [7] conducted a survey of medical students at all medical schools in Canada. The response rate was 40.2% or 4613 out of the 11,469 medical students. The survey found that medical students had higher rates of mental illness than the general population. Importantly, 14.6% had suicidal ideation, with 6.1% of the participants having had suicidal thoughts in the last twelve months. The study also found that female medical students had higher rates of mood and anxiety disorders, moderate or severe psychological distress, and overall suicidal ideation. Interestingly, clinical years appeared to be a higher risk period. As the study revealed, medical students had higher rates of suicidal ideation, moderate or severe psychological distress, and mood and anxiety disorders during clinical training.

Karp and Levine [3] explained that the reasons for the high rates of mental illness among medical students is related to the demands of a medical degree such as financial cost and stigma. Billingsley [4] also pinpointed the demands of medical education and perceived stigma as the basic culprits. Slavin, Schindler, and Chibnall [8] showed that stigma was an important barrier for medical students, and Janoušková et al. [9] explained that stigma was a main factor for keeping medical students away from talking about or reporting mental illness. Furthermore, the same author suggested that their stigmatising attitudes changed when they learnt more about mental illness during their psychiatry rotation. Along similar lines, Azim [5] discussed that stigma has been a barrier to recognising mental illness and seeking appropriate help. Moreover, several factors contributing to the high rates of mental illness among medical students were outlined, including: competition, academic demands, social expectations, and financial problems. In support, Brazeau et al.’s study [10] indicated that medical students had better or similar mental health than the general population when first entering a medical programme and that their mental health deteriorated over time while being a medical student.

Drawing evidence from the literature, Constantinou, Georgiades, and Papageorgiou [11] suggested the PEACE guidelines in order to enhance medical students’ psychological wellbeing. PEACE stands for Professional counselling and support structures, Engagement with social activities, Active mind and psychological wellbeing, and Curriculum Efficiency. The authors explained that professional counselling and support structures were largely for the time when students needed help and suggested that, apart from professional counselling, the training of both faculty and staff was essential for helping medical students. Eisenberg, Hunt, and Speer [12] had highlighted the importance of gatekeeper training in recognising students’ psychological symptoms and providing support. Although gatekeeper is a broad term and can include many groups in a community, it includes members of faculty and staff. Moreover, Azim proposed solutions for tackling the high rates of mental illness among medical students that were largely on par with Constantinou, Georgiades, and Papageorgiou’s approach. Those included student-centred learning pedagogies, timely spaced exams, early engagement with clinical practice, communication skills, and extracurricular activities. Azim [5] placed emphasis on the support systems, maintaining that recognising students’ symptoms was not enough and that referring students to the appropriate structures for personalised support was of paramount importance.

Considering the high rates of mental illness among medical students, the need to ensure that highly competent clinicians are psychologically well equipped to service the community, and the suggestion that faculty and staff could be trained to support their students, this study had two objectives: (a) to explore whether faculty and staff at medical schools have been involved in this type of student support, and (b) to explore any available training for faculty and staff in how to recognise medical students’ psychological symptoms and provide support, such as referrals and basic advice. We focused on members of faculty (i.e., teachers) and staff (i.e., administrative and other non-teaching staff) as these two groups work closely with medical students. We generated these two objectives and determined the most suitable type of review and methodology by following Maier’s [13] pathway as described below. 

## 2. Methodology

As per Maier’s pathway [13] we started with the “identification of the problem domain” by reading the basic literature. From the initial search of the literature, we realised that there were not many studies on training of faculty and staff in recognising medical students’ psychological symptoms and therefore we decided that a narrative literature review was more appropriate than other types of review such as systematic or meta-analysis. In support of our decision, according to Paré and Kitsiou [14], a narrative review should aim to summarise and synthesise what has been written on a subject area. Also, narrative reviews are particularly useful for identifying gaps in knowledge and, as a result, inspiring further research [15].

After identifying the knowledge gaps and deciding the type of review, we then “critically discussed what has been done” [13] by searching, selecting and reviewing the existing literature on the subject matter. From the review of selected articles, we “identified knowledge gaps” and generated more “objectives” [13] by formulating suggestions and recommendations for future practice and research. 

Having decided that a narrative literature review would be more suitable for addressing our objectives, we relied on guidelines by Ferrari [15] and the SANRA scale (Scale for the Assessment of Narrative Review Articles) [16] for selecting and reviewing articles. These guidelines clarified that narrative reviews should include a clear aim, justification, and a search strategy. The objective of this narrative review was to explore the involvement of faculty and staff in recognizing medical students’ psychological symptoms and providing support, and any training. Due to the lack of a fixed research hypothesis, as per the guidelines for narrative reviews [15], our inclusion and exclusion criteria were as per Table 1. Here, it is important to clarify that we did not aim to review the literature in mental health first-aid programmes, as these have already been reviewed by other scholars. Also, mental health aid programmes are lengthy training, and they involve learning skills in how to respond during mental health crises. This study focused on training regarding recognising symptoms and providing support such as referrals and basic advice. However, in our keywords we added “mental health first aid” to identify any training for faculty and staff in medical schools that had shorter components that could reflect the objectives of our study.

Based on the criteria above, the following databases were searched: Scopus, Web of Science, Embase, PubMed, Medline, Google Scholar, PsycInfo, and EBSCO. In order to achieve a focused search and address the objectives of the study, we used specific keywords, and these were: psychological symptoms, mental health first aid, training, evaluation, faculty, teachers, staff, administrators, gatekeepers, support, medical students, undergraduate medical programmes, healthcare, university, college, tertiary education, higher education, students, depression, suicide, anxiety, stress. For facilitating our search and review of the identified articles we used specific questions as per Table 2.

We did not treat the questions in Table 2 as research questions but only as guides for our search and for reviewing the articles in order to ensure that the articles selected were relevant. These questions were also used as a context for generating codes and constructing overarching themes. 

**Figure 1:**
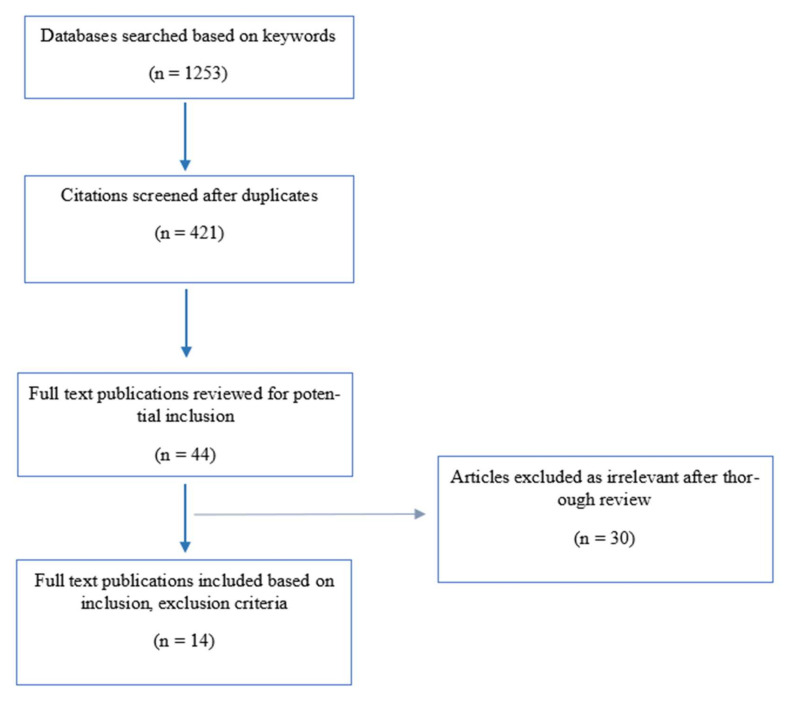
Flowchart on the literature selection process based on guidelines by Ferrari [13].

As per Figure 1, the initial search generated 1253 articles. The process of excluding duplicates and irrelevant papers resulted in 421 articles. Based on reading the abstracts of these sources in accordance with our inclusion and exclusion criteria, 44 sources were selected for in-depth review. The detailed review resulted in the selection of 14 articles as they reflected, directly or indirectly, the objectives of this study.

For the analysis of the articles, we relied on an interpretive approach to fully understand the effectiveness of any training for medical faculty and staff and on Thomas and Harden’s [17] thematic synthesis technique. Thematic synthesis consists of three stages. Firstly, the articles were thoroughly read multiple times to become familiar with the methods and the findings. Secondly, the findings in each article were coded based on the objectives of this study. Thirdly, the codes were grouped together in order to construct overarching themes, ultimately forming a codebook that helped organise and interpret the results. After the construction of the codes and themes, the analysis was drafted and refined by revisiting the articles, initial codes, and themes. To ensure the validity of the results, a two-level quality assurance process was instituted, whereby the researchers split into two groups and followed the review procedure (check codes and themes, revisit the articles, refine the codes and themes) independently.

For the critical appraisal of the articles included, we used the criteria as outlined by Ferrari [15], namely key results, limitations, suitability of the methods used to test initial hypothesis/aims, quality of results obtained, interpretation of results, and impact of the conclusions on the field. The critical appraisal of the articles is presented under Section 4 in the form of a narrative text, and in a detailed table.

## 3. Results

Interestingly, there were no studies of training involving members of faculty and staff at medical schools that aimed at helping trainees to recognise students’ psychological symptoms and provide support (i.e., referrals or basic advice), identified within this review. Although there were many programmes involving medical students as trainees, we were not able to identify any training tailored for faculty and staff in a medical programme. In addition, our search included ‘mental health first aid programmes’ in order to observe if there were any early components on recognising medical students’ psychological symptoms. However, we did not find any studies describing and evaluating mental health first-aid programmes tailored for medical faculty and staff. 

The lack of training for faculty and staff at medical schools is an important finding because, as already highlighted in the introduction of this article, there is strong evidence to suggest that mental illness among medical students is higher than that of the general population and among students of other disciplines. This review, however, did find articles that pertained to faculty and staff in higher education that could shed light in developments for medical schools as well. From the coding of these articles, three overarching themes emerged. That is, “faculty/staff’s awareness and acknowledged importance of recognising students’ psychological problems”, “faculty/staff’s perceived preparedness”, and “the effectiveness of training”.

### 3.1. Faculty/Staff’s Awareness and Acknowledged Importance of Recognising Students’ Psychological Problems

All articles reviewed indicated that faculty and staff acknowledged the importance of recognising students’ psychological symptoms and providing all necessary support. This acknowledgement largely derived from faculty and staff having been aware that university students were likely to experience mental illness or problems and that mental wellbeing was essential for students’ health and academic success [18,19,20,21,22,23]. For example, a large longitudinal survey by Sontag-Padilla et al. [19] in the California Community College with the participation of 14 campuses showed that faculty and staff were concerned about their students’ psychological wellbeing. That is, the surveys took place in 2013, 2014, and 2017 with 942, 812, and 1132 participating faculty and staff, respectively. Nearly 70% of participants reported that they were concerned about the mental health status of at least one of their students, while many of these participants provided the necessary support to the students. Around 70% of them had talked with at least a student with mental health problems, and 51% referred their students to mental health support services. Similar trends were reported by Margrove, Gustowska, and Gorve [19] in their study of administrative staff at two UK universities.

Interestingly, faculty and staff with experience in working with students who had mental health challenges appeared to feel responsible and willing to help their students. Kalkbrenner et al. [18] showed that faculty and staff expressed their willingness to recognise symptoms and refer their students to the appropriate services, and Sontag-Padilla et al. [19] discussed how faculty and staff were positive about the available mental health services at the university. Spear, Morey, and van Steen [24] revealed that most participants in their study encountered students with mental disorders, although only 56% of the participants had some training in order to handle cases. Moreover, Sylvara and Mandracchia’s study [25] focused on understanding gatekeepers’ training and self-efficacy for suicide intervention and approached 3700 faculty from various higher education institutions in order to complete a survey, which was eventually filled in by 507 members of faculty. The results showed that the majority of participants thought that faculty members had the responsibility to recognise their students’ psychological symptoms and identify the students who were at risk of suicide. Also, a study by Lispon et al. [26] indicated that faculty and staff understood that their students’ mental health had worsened and that universities should invest more in supporting their students. Lipson et al. explained that 80% of their participants had had some form of communication with students about their mental health in the last 12 months. 

From the studies above it transpires that the need to recognise students’ psychological symptoms and provide support has been well established. It is interesting to see how prepared the faculty and staff are for this task, as presented below. 

### 3.2. Faculty/Staff’s Perceived Preparedness

Interestingly, although studies generally agreed that faculty and staff were aware of the magnitude of the problem, they acknowledged their own responsibility in providing help, and were willing to support their students, they had clearly expressed the view that they were not well prepared, and that training was imperative. The review of the selected articles showed that faculty and staff had limited knowledge about the resources and what some services really offered, and that sometimes they did not know how to successfully recognise their students’ psychological symptoms. Sontag-Padilla et al.’s [19] study of faculty and staff’s perceptions in California Community College campuses indicated lack of preparedness as not much training was offered by their institution. Interestingly, in the six months before the study only 25% of faculty and staff took part in relevant training. More specifically, 41% of faculty and staff felt confident to help students with psychological symptoms, and 56% were comfortable to discuss mental illness issues with their students. 

More studies indicated that faculty and staff expressed the view that they were not well prepared to recognise their students’ psychological symptoms and take appropriate action to help their students. For example, Margrove, Gustowska, and Grove’s [21] research revealed that more than 70% of their participants were untrained, whereas almost 40% of them could not distinguish between daily challenges and psychological symptoms. Spear, Morey, and van Steen’s study [24] indicated that only 31% of the participants felt that their university prepared them adequately for recognising students’ psychological symptoms, whereas Lipson et al. [26] found that only 51% of faculty members felt confident in how to recognise their students’ psychological symptoms. In addition to lack of confidence and experience, the study found that the participants who worked with students with mental health problems had other difficulties that made their involvement challenging. That is, they experienced disruption to other students, inappropriate behaviour and communication, unrealistic complaints, and sometimes threatening behaviour by students with mental health problems. Lipson et al. [26] showed that 51% of their participants reported knowing how to distinguish between emotion and mental distress, whereas only 29% said that they knew how to tell that a student showed signs of substance abuse. Because of perceived unpreparedness, 73% supported more training and 61% thought that training should be mandatory for all faculty members. 

Another factor affecting preparedness found in the literature was that of perceived stigma and prior knowledge in mental health problems. Gulliver et al. [27] recruited 224 academic staff to understand their knowledge and attitudes about mental health issues and how these knowledge and attitudes affected how they helped their students. The results showed that female participants and those with a health and behavioural science background scored higher in the depression literacy scale. Importantly, those who scored higher in the depression literacy scale had significantly lower scores in the stigmatising attitudes scale. Also, those who knew more were more likely to be involved in a conversation with a student regarding the student’s mental state. 

All articles that discussed that faculty and staff were not well equipped to recognise their students’ psychological symptoms highlighted the importance of the need for more formal guidance and training. It is important to see what training has been offered and whether it helped faculty and staff, as discussed below.

### 3.3. The Effectiveness of Training

In the articles reviewed, there was no training for recognising students’ psychological symptoms in general, and for providing support, such as referral and basic advice. However, the review generated four studies that evaluated the effectiveness of training on preventing suicide. These trainings were effective in increasing awareness and helping trainees gain confidence and skills. Hashimoto et al. [28] aimed to evaluate a 2.5 h programme on suicide prevention. The authors recruited 76 administrative staff at the Hokkaido University in Japan. Any changes were measured by a self-reported questionnaire before the training, immediately after, and a month later. The results revealed that the programme helped trainees gain more confidence and competence in working with students with suicidal thoughts. The changes elicited by the training continued to be observed a month later. The training also helped trainees to maintain their interest and intention to help students. Hashimoto et al.’s [29] follow-up study with university teachers yielded similar conclusions.

Zinzow et al. [30] evaluated the effectiveness of a 90 min training programme. The programme trained 517 students, faculty, and staff and was longitudinal in the sense that it involved a pre-test/post-test process and a follow-up three months later. The training programme helped faculty and staff to improve their knowledge and their self-efficacy in discussing suicide and referring students to the right structures. Along similar lines, the conclusion from Sylvara and Mandracchia’s [25] evaluation was that members of faculty who had training were more confident and enhanced their skills regarding helping suicidal students. 

## 4. Critical Appraisal of the Articles Reviewed

Critical appraisal of the selected articles was carried out in accordance with Ferrari’s [15] guidelines (see Table 3 below). Ferrari presented six criteria for articles included in narrative reviews. They are, key results, limitations, suitability used to test initial hypothesis/aim, quality of results obtained, interpretations of the results, and impact of the conclusion on the field.

All articles had summaries of their key findings and the results were discussed adequately. The articles that presented studies had a clear aim and employed relevant study designs. With the exception of a phenomenological study by Kalkbrenner, Jolley, and Hays [18] and part of the study by Margrove, Gutowska, and Grove [21], the rest employed quantitative methodologies, largely surveys. There was no study, even the ones that evaluated training programmes [25,28,29,30], that relied on a randomised controlled trial. Although the methodologies used were appropriate for addressing the study aim, two limitations accrued from the set of articles reviewed. First, more qualitative studies would help understand in more depth the complexity of the issue of mental illness among higher education students, the needs, the content of training, and also their effectiveness. Second, randomised control trials would have provided more insights into the impact of training programmes in the trainees’ knowledge, skills, and attitudes, establishing greater confidence in how useful and effective the training would be. 

The results of the articles were of good quality and related to the aim of the studies. More specifically, the qualitative studies [18,21] presented data in the form of interpretive text, studies that employed quantitative methodologies [19,21,24,27] presented descriptive statistics, correlations, and power relations, whereas discussion papers [5,12,20,23] reviewed approaches and discussed them more thoroughly. The results were discussed appropriately, and research articles presented limitations and future directions [19,21,24,27,28,29]. Importantly, all articles were well justified and explained their contribution to scholarship. For example, the research articles [19,21,24,27,28,29] explained that their study was needed in order to fill in an identified gap in the literature, whereas discussion papers focused more on putting various approaches together and opening new directions [5,12,20,23].

## 5. Discussion

This narrative literature review explored articles published between 2000 and 2021 that focused on the training of faculty and staff in recognising medical students’ psychological symptoms and providing support, such as referral and basic advice. This narrative review did not aim to include studies of mental health first-aid programmes but only training that was shorter and broader in focus and did not relate to response during crises. The results did not show any training in medical education, which we found striking because mental illness among medical students is higher than among students from other disciplines. However, we included articles that related to higher education students. The results indicate that faculty and staff tended to consider the matter important, that they would like to be trained and provide all necessary support to their students, such as basic guidance and referral, and that members of faculty and staff should be involved in this type of support. 

Other studies on the mental illness of medical students as outlined in the introduction of this article seemed to support the findings of this narrative review. That is, Rotenstein et al. [2] showed the prevalence of mental illness among medical students explaining the reasons why this is happening, whereas Constantinou, Georgiades, and Papageorgiou [11] highlighted the importance of a more holistic approach to the mental illness of medical students, including the training of faculty and staff. Moreover, Janoušková et al. [9] placed emphasis on perceived stigma as a barrier to seeking help for medical students. Interestingly, this is not only a barrier for medical students but it can be potentially for faculty and staff, and this narrative review has surfaced it. The study by Gulliver et al. [24], included in this review, showed that perceived stigma could not be a barrier only for students, but also for faculty and staff, keeping them away from being involved in discussions with students about mental illness; interestingly, the less faculty and staff knew about depression the more likely it was the perceived stigma to be a barrier. This narrative review also indicated that faculty and staff were not well prepared to work with students in need and support them appropriately and that training seemed to be effective in terms of helping trainees gain more knowledge and enhance their skills and confidence in working with student with mental illness. This finding supports the call by other studies or reports for more investment or efforts to train students, faculty, and staff [5,11,12].

A striking finding of this narrative review is that we did not identify any training for medical schools’ members of faculty and staff. This is in fact a worrying finding because the literature shows that medial students have the highest rates of mental illness, largely due to the demands of a medical degree, perceived stigma, and financial cost [3,5]. On this note, it is imperative to consider the following directions in research:

- What are the reasons for the high mental illness among medical students?
- What are the mental health needs of medical students and what training for faculty and staff can address students’ needs?
- Is this training effective? Does it help faculty and staff gain confidence and skills in order to recognise students’ psychological symptoms and provide support?
- Does the training help students’ deal with their psychological symptoms and help them develop as learners and professionals later on?

### The CReATE Circle

**Figure 2:**
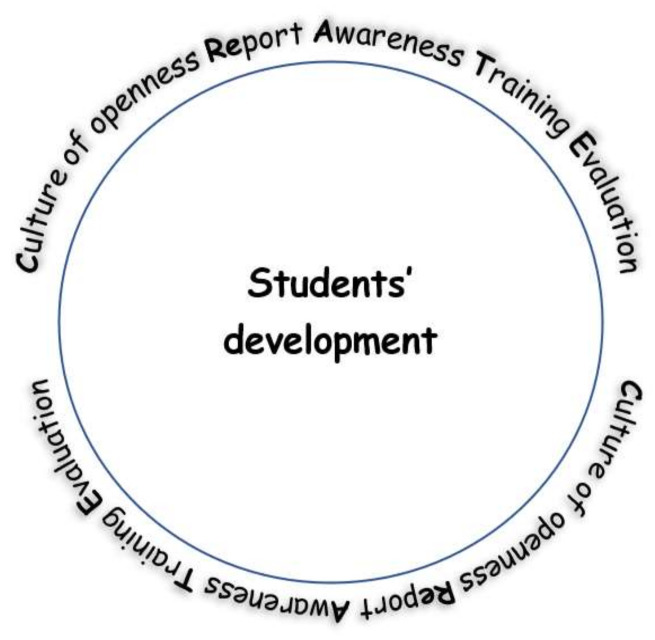
The CReATE circle for recognising students’ psychological symptoms, provide support and help students develop.

Having discussed the results from the narrative review and considered the new research directions opened, we propose the CReATE circle in order to successfully recognise students’ psychological symptoms, provide necessary support, and help students develop. As per Figure 2 below, CReATE stands for **C**ulture of openness, **Re**port, **A**wareness, **T**raining, **E**valuation. **C**ulture of openness is necessary for both students and faculty/staff to understand that mental illness is not rare, it can affect everybody, and it is to a large extent manageable and reversible. Such openness can help to deal with perceived stigma, which is very often a barrier for both students, and faculty and staff. As a result, students, faculty and staff would be more comfortable to **Re**port symptoms and make the necessary referrals. **A**wareness of the available structures of support is essential in order to know where to go or refer to when crisis strikes. **T**raining of faculty and staff but also of students is important and should be lifelong instead of one-off or ad hoc workshops. The last component of the circle is **E**valuation of training, but also of students’ mental state by monitoring the numbers of students who experience mental illness symptoms and how they develop in the future. The five components of CReATE should be in a circular relationship including review, reflection, and continuous enhancement in order to make sure that the quality of support is high and the structures are sustainable. CReATE phonetically resembles the word “create” to place emphasis on the importance of creating the necessary procedures for successfully recognising students’ psychological symptoms and supporting them appropriately in a sustainable manner. 

The CReATE circle reflects the theoretical underpinnings of the PEACE guidelines for enhancing psychological support of medical students [11], as well as creating a culture of openness and practice at an institutional level [31]. Constantinou et al. [11] highlighted the importance of structures of support, information and training, and promotion of psychological wellbeing that could enhance a culture of openness and reporting medical students’ psychological symptoms and providing support. Echoing Martins and Martins [31], such a culture of openness would be achieved more efficiently when all parties involved shared the same vision, objectives, and pathways of collaboration. As a result, faculty, staff, and students would work as a team to achieve the same goal: that of enhancing students’ psychological wellbeing and ensuring the graduation of highly competent clinicians. 

## 6. Conclusions

This narrative literature review explored published work on training faculty and staff in recognising psychological symptoms of medical students and providing the necessary support. The review explored 14 articles that met the inclusion criteria and has made an important contribution to scholarship by finding that no such training has been identified in medical education, although the rate of mental illness among medical students is very high. Reviewing the articles that were relevant to higher education, we concluded that the need for faculty and staff to recognise their students’ psychological symptoms has been acknowledged, faculty and staff have largely been unprepared for this task, any relevant training seemed beneficial, and the need for training faculty and staff has been well established. On this note, with this review we propose the CReATE circular pathway, whereby a culture of openness is needed that can help reporting of psychological issues and seeking help, awareness of the support structures should be enabled and enhanced, and training and evaluation of this training, as well as the whole system of support, should be ensured. This review has also established that there is a big gap in researching the content and effectiveness of training tailored for faculty and staff at medical schools.

## Figures and Tables

**Table 1:** Inclusion and exclusion criteria.

Inclusion	Peer-reviewed articles or chapters
	Theses and dissertations
	Literature reviews
	Conference papers
	Editorials
	Books or textbooks
	All of the above that include or discuss training of faculty and staff in recognising students’ psychological symptoms in tertiary education
	Published in English
	Period of publication: 2000–2021
Exclusion	Peer-review articles, chapters, theses or dissertations, conference papers, editorials, books and textbooks that do not include or discuss training of faculty and staff in recognising students’ psychological symptoms in tertiary education and providing support
	Any of the above publications published before 2000
	Published in languages other than English

**Table 2:** Questions for facilitating search strategy.

Has the need for training faculty and staff in recognising undergraduate medical students’ psychological symptoms and providing support been assessed and documented?
What are the faculty/staff’s views on recognising medical students’ psychological symptoms and providing support?
Is there any training of faculty and staff in recognising undergraduate medical students’ psychological symptoms and providing support? Is there such training in other disciplines in tertiary education?
What did this training entail?
Has this training been effective in terms of enhancing faculty and staff’s skills and confidence?
Has this training been effective in terms of identifying psychological symptoms early and helping students?
Has this training shown any long-term effects?

**Table 3:** Critical appraisal of articles reviewed based on Ferrari’s [15] criteria and guidelines.

References Number	Article	Key Results	Limitations	Suitability of the Methods Used to Test the Initial Hypothesis/Aims	Quality of the Results Obtained	Interpretations of the Results	Impact of the Conclusions on the Field
[12]	Eisenberg, D., Hunt, J., & Speer, N. (2012). Help Seeking for Mental Health on College Campuses: Review of Evidence and Next Steps for Research and Practice. Harvard Review Of Psychiatry, 20(4), 222–232. https://doi.org/10.3109/10673229.2012.712839	Most college students with mental health problems are not receiving treatment. Wide range of factors influence students’ help seeking for mental illnesses; traditional barriers (knowledge and stigma) not the only reasons (many untreated students do not have deep-rooted attitudes that prevent them from receiving treatment). Most common intervention strategies to address help seeking on college campuses categorized into three groups: - stigma reduction and education campaigns - screening and linkage programs - gatekeeper training	It is not clear what methodology has been used for searching and reviewing the evidence.	Review study addresses the study objective: advance our thinking about how to increase use of appropriate services among college students with significant mental health problems.	Results are well presented and reveal what is known about help-seeking behaviour and what can be done in the future to increase the number of students who receive intervention.	New strategies may prove to be important for changing behaviour of large numbers of students who are not using services. Seeking help for mental health involves short-term costs (e.g., time, energy, money) with expectation of better health in future.	New approaches to help seeking in college setting need to be explored. More effective overall set of strategies will yield great benefits to young people (will also benefit society).
[18]	Kalkbrenner, M.T., Jolley, A.L. and Hays, D.G., 2019. Faculty views on college student mental health: Implications for retention and student success. Journal of College Student Retention: Research, Theory & Practice, p.1521025119867639.	Knowledge of MHD definition as a combination of genetic and environmental factors. Knowledge of warning signs as changes in behaviour. Comfort and willingness to recognize and refer was impacted by personal experiences and the stigma in college environment. Limited knowledge about resources for mental health issues (not specifically aware of services provided or philosophies held by college counselling centre). Faculty-student relationships influenced level of comfort associated with reaching out to students about MHDs and also the classroom context (harder to recognize MHDs in large lecture hall).	Results may not be generalisable to all faculty members. Security of tenure amongst two participants may have influenced their comfort in supporting student mental health. Three participants trained in mental-health- related fields that may have influenced their perceptions on MHDs. Self-selection bias. Snowball sampling procedure (recruiting participants from similar academic departments).	Phenomenological Study: Allowed researchers to explore the experiences of faculty members and address the two research questions: - How do faculty members who have lived experiences with supporting college student mental health conceptualize MHDs among college students? - What contextual variables relate to their likelihood to refer students with mental health symptoms to university resources?	Qualitative Data: Explores more in-depth how faculty members conceptualize and recognize MHDs, along with factors that influence the degree to which they provide accommodations. Results clearly presented in five emergent themes that addressed the research questions.	Faculty knowledge of MHDs and warning signs (behaviour changes) showcases the importance of utilizing faculty members as resources to identify students with MHDs who might not seek out services. Limited knowledge about resources for mental health issues suggests that faculty members have an ade quate awareness of the general availability of university resources; however, many did not have knowledge about specific services	Faculty have a responsibility to support student mental health. Recommended: - Awareness and Education about College Counselling Centre - Unification of Campus Resources and Policy - Structuring the Academic Environment - Advocacy for College Counselling Centres
[19]	Sontag-Padilla, L., Dunbar, M.S., Seelam, R., Kase, C.A., Setodji, C.M. and Stein, B.D., 2018. California community college faculty and staff help address student mental health issues. Rand health quarterly, 8(2).	Community college students experience mental health challenges and adverse circumstances (e.g., homelessness) that put them at risk for ongoing problems. Most faculty and staff acknowledge concerns about the mental health of students on their campuses, and many take action to help students with mental health needs. Most faculty and staff surveyed in 2017 held favourable views of their campuses’ student mental health services, with positive perceptions increasing since 2013. Just over half of faculty and staff surveyed reported that their campuses are actively putting into place training programs to help faculty and staff recognise and respond to students with mental health needs. In the six months prior to the survey, only a quarter of faculty and staff participated in training on how to better support students with mental health problems. Continued efforts are needed to ensure that faculty and staff are equipped to address student mental health issues on campus.	Selection Bias (Not all campuses invited all faculty and staff to participate). Self-Reported Data (No objective information).	Survey collecting quantitative data in regard to the knowledge, attitudes, and behaviours of faculty and staff in regard to supporting mental health needs of students.	Results of the survey addressed all three aims: - Campus experiences and attitudes related to student mental health - Perceptions of how campuses are serving students’ mental health needs - Perceptions of the overall campus climate toward student mental health and wellbeing Promising findings but still shows that there is room for improvements for faculty and staff. Results clearly presented in tables.	Most faculty and staff acknowledge concerns about mental health of students on campuses and take action to help students with mental health needs. Most staff had concerns about their ability to help students, hence suggesting that they need to continue to provide staff with resources so they can become more confident in helping their students.	Faculty and staff members should participate in training (online seminars, individualized programs, group sessions) to ensure they feel more confident in their abilities to help students with their mental health needs.
[20]	Cook, L.J., 2007. Striving to help college students with mental health issues. Journal of Psychosocial Nursing and Mental Health Services, 45(4), pp. 40–44.	The severity and number of mental health problems is increasing among college students across the United States. Psychiatric mental health nursing faculty can work to develop support systems for students with mental health problems through the establishment of the National Alliance on Mental Illness (NAMI) on campus programs, which provide advocacy, education, and support to students with mental health issues. Psychiatric mental health nursing faculty can incorporate NAMI activities and suicide awareness and prevention programs for the college campus into various courses that address mental health issues and involve faculty and students from other disciplines.Seeking grants to develop education, advocacy, and student support activities is another way psychiatric nursing faculty can improve mental health programs on college campuses.	Viewpoint of the efforts of one psychiatric mental health nursing faculty member.	Review of programs to help reach students with mental health needs through establishment of on-campus NAMI through discussion of establishment goals and past accomplishments to help.	Through discussion of research articles, the author showcases the increasing need of services to address mental health needs.	Mental health nursing faculty can help address problems on college campuses by offering courses on mental health issues and skills.	Mental health problems in college are increasing in number and severity and many people are not willing to seek help, there is a need for additional staff and programs that are able to help students in need.
[21]	Margrove, K., Gustowska, M., & Grove, L. (2012). Provision of support for psychological distress by university staff, and receptiveness to mental health training. Journal Of Further And Higher Education, 38(1), 90–106. https://doi.org/10.1080/0309877x.2012.699518	Most staff participating in this research had experience of providing support for psychological distress. Over half of the staff participants indicated that they felt more training in mental health could benefit them. Most survey respondents were aware of signs of major depression and schizophrenia, and many could detect that normal troubles were not likely to be an indication of a mental health problem.	Research confined to staff from one type of faculty. Participants’ views about what constitutes psychological distress may have varied.	Anonymous online survey to identify whether staff provided support for psychological distress to students and colleagues and to see whether staff are trained in mental health issues. Survey included optional free-text space allowing participants chance to comment on mental health training in workplace (included in case participants wanted to say something that was not addressed by other quantitative questions).	Both quantitative and qualitative results presented clearly and addressed research aims.	High levels of psychological distress in students could be placing additional demands on university staff working in both administrative/support or academic roles.	Higher education institutions should introduce training for their employees with experienced and qualified mental health training providers. Further qualitative research should be completed to help shed light on issues in greater depth.
[22]	Ardekani, A. et al., 2021. Student support systems for undergraduate medical students during the COVID-19 pandemic: A systematic narrative review of the literature. *BMC Medical Education*, 21(1).	“Taken together, the results of our review assert that methods of supporting medical students should be adapted to the new circumstances and environments and should provide different levels of support through both online and in-person strategies”	There are no randomized control trials or high-quality interventions on the efficiency of support systems devised in response to COVID-19. There are currently a small number of studies that fit the research criteria. The included studies focused on describing their methods rather than evaluating the outcomes.	The studies included were reviewed and underwent critical appraisal. Though none of the chosen studies were excluded after being found to be low quality.	None of the included studies met the criteria (7 out of 11 items checked against the Buckly et al. criteria) to be considered high-quality studies.	Most studies tend to describe the educational intervention rather than evaluating its outcomes. Methods of supporting students should be adapted to new circumstances and environments. Support should be offered through both in person and online strategies	Future research should focus on evaluating the outcomes of intervention strategies.
[23]	Rau, T., Plener, P., Kliemann, A., Fegert, J. M., & Allroggen, M. (2013). Suicidality among medical students–a practical guide for staff members in medical schools. GMS Zeitschrift fur medizinische Ausbildung, 30(4), Doc48.	Risk factors for suicidal behaviour among medical students—fear, depression, negative life experiences, impulsivity, female gender, physical discomfort, low SES, low quality of life, perceived level of stress, developmental crises, and difficulties separating from parents. Indicators of increased suicide risk—presenting with suicidal thoughts, risk factors, current stressful situations, psychopathological conspicuities that show themselves as hopelessness, fear, anxiousness, irritation. Recognizing suicidality by staff members—students expressing feelings of being overwhelmed, pressured, overburdened, radiate hopelessness, sadness, abrupt change in behaviour, increased absence. Staff should approach the topic of suicidality in the context of a conversation, show understanding, signal willingness to support, offer help/assistance where possible. Have additional meetings set up. Scenarios: Suicidal thoughts are not yet present but when student’s express feelings of excessive stress and hopelessness—professional help Expressing suicidal thoughts—specific and immediate risk needs to be decided. Intentions to act are being communicated— immediately seen by psychiatrist, ER Providing expert training workshops— Participants showed significant increase in their level of knowledge and confidence in their own competence to act when pre- and post-training data were compared.	Literature review—causation cannot be made. Assessing suicidality in different studies that have differing methodological approaches, composition of samples (age, year of study, gender proportionality), and cultural context. Confounding variables—complex interplay between factors	Literature review. Aims—describe the epidemiology and factors leading to suicidality in medical students and demonstrate options for handling suicidal crises in students.	Article search published between 1993–2013 via Ovid using the data bases Medlin and PsycINFO.	Suicidal thoughts are more prevalent among medical students than in the general population of comparable age group. Staff members should have adequate training and gain basic knowledge and skills in dealing with the issue of suicidality to be better prepared and have increased confidence and competence in confronting risk situations. Expert training enables staff to become more sensitive in recognizing and dealing with at risk students and to provide them with practical competences to act effectively in such situations.	Helps raise awareness of suicidality among students, demonstrate effective strategies, and training needed for handling suicidal crises. Such training programs can provide a crucial contribution to suicide prevention at universities.
[24]	Spear, S., Morey, Y. & van Steen, T., 2020. Academics’ perceptions and experiences of working with students with mental health problems: Insights from across the UK higher education sector. *Higher Education Research & Development*, 40(5), pp.1117–1130.	Nearly all respondents (96%) had encountered student mental health problems (MHP) amongst their students (n = 130). This was true across each institution type: Russell group—92%, pre-1992 group—97%, post 1992 group—98% Respondents’ awareness of student MHPs was high but the preparedness to support students was low. 31% felt their current institution adequately prepared them for working with students. 56% had not received training in working with students MHP.	A limited number of respondents and the self-selecting nature of the survey selected for participants that already had some interest in student mental health. It did not allow for a reach to staff with less interest, which would have given a more holistic understanding of staff perception and student experiences. The study focuses on academics with any teaching or supervision responsibilities. A more focused study on specific student groups and academics would highlight problems those groups face. There was a low response rate from institutions and academics, so it is not a representative sample of UK universities. The study focused on mental health problems in one national context (UK).	The use of a quantitative first phase and a qualitive second phase allowed for a more comprehensive investigation into the main study aim.	The sample size of the study was small.	96% of all the staff that responded to the survey had encountered MHP in their students. Students often turn to academic staff for initial and ongoing support as they have most contact with these staff and are likely to trust them most.	Academic staff should be an integral part of any institutions strategy for enhancing student mental health.
[25]	Sylvara, A. L., & Mandracchia, J. T. (2019). An investigation of gatekeeper training and self-efficacy for suicide intervention among college/university faculty. *Crisis*.	Most participants reported believing it is the college/university faculty’s role to identify students at risk for suicide; however, many reported that their institution did not provide gatekeeper training. Participants who had received gatekeeper training were more confident in identifying and assisting at-risk students.	The study did not include demographics (sex, type of institution, department of faculty, state of residence). Participants knowledge of their institutions’ policies and procedures relating to referring and intervening with a student who presents as suicidal (which was the purpose of the study) was not directly evaluated but the participants belief on how familiar they were with the policies was evaluated instead. The participants were not asked whether they had been trained in suicide prevention or intervention before.	The methods accurately investigated the study’s aims.	The study used 6 *t*-tests to assess the tested outcomes. The results were accurately assessed and proved to be statistically significant were reported.	Faculty should be trained in assisting at-risk students so that they can develop more confidence and better refer at-risk students.	Training faculty to assess and respond to at-risk students may decrease suicide deaths among college/university students.
[26]	Lipson, S. K., Talaski, A., Cesare, N., Malpiede, M., & Humphrey, D. (2021). The role of faculty in student mental health. *Boston University, Mary Christie Foundation and The Healthy Minds Network*.	87% of faculty believe that student mental health has “worsened” or “significantly worsened” during the COVID-19 pandemic. Almost 80% have had one-on-one phone, video, or email conversations with students in the past 12 months regarding student mental health and wellness. Only 51% of faculty reported that they have a good idea of how to recognize that a student is in emotional or mental distress. 73% would welcome additional professional development on the topic of student mental health. 61% believe it should be mandatory that all faculty receive basic training in how to respond to students experiencing mental or emotional distress. 21% of faculty agree that supporting students in mental and emotional distress has taken a toll on their own mental health. Half believe their institution should invest more in supporting faculty mental health and wellbeing.	Selection bias—university staff members self-selected to participate in the study.	Aim—understanding faculty members’ perceptions of student mental health needs, faculty’s experiences supporting students, and the need for institutional resources to address both student and faculty mental health.	Survey responses from 1685 faculty members at 12 colleges and universities across the United States	Majority of faculty members would welcome more training in how to support students experiencing mental health issues and believe that this training should be mandatory.	Universities can do a better job in supporting faculty in addressing the mental health of all students.
[27]	Gulliver, A., Farrer, L., Bennett, K. and Griffiths, K., 2017. University staff mental health literacy, stigma and their experience of students with mental health problems. Journal of Further and Higher Education, 43(3), pp. 434–442.	Teachers with higher levels of depression literacy were more likely to engage with students with mental health problems and felt sufficiently informed to help. Higher levels of stigmatising attitudes to depression did not independently predict whether a teaching staff member would approach a student to assist with a mental health problem. Stigmatising attitude did not impact on a staff member’s willingness to approach a student to assist with mental health problems.	Selection bias—university staff members self-selected to participate in the study. The sample comprised a single university, and thus may not be representative of all university staff. Only attitudes and knowledge related to one common mental disorder were assessed (depression), staff could have had more knowledge of or demonstrated more stigmatising attitudes to other mental health problems.	Aim—to identify if university staff attitudes to and knowledge about mental health problems, or whether these factors influence their experience with and assistance of students with mental health problems. Measures—university staff literacy, the stigma they attach to depression, and the influence of these factors on their experience with and assistance of students with mental health problems.	224 teaching staff members at Canberra University completed an anonymous online survey via an email link that featured a series of questions adapted from Reinke et al. (2011) involving— demographics, professional information, experiences with student mental health, knowledge of depression (literacy) and attitudes to depression (stigma).	University staff may be unlikely to allow personal beliefs to interfere with their professional judgement.	Ensuring staff complete mental health literacy training and have adequate skills to respond appropriately to students with mental health problems may help in connecting young people to appropriate care in a university context. University staff members are well positioned to offer an initial point of contact for referral to appropriate sources of professional help.
[28]	Hashimoto, N., Suzuki, Y., Kato, T. A., Fujisawa, D., Sato, R., Aoyama-Uehara, K., … & Otsuka, K. (2016). Effectiveness of suicide prevention gatekeeper-training for university administrative staff in Japan. *Psychiatry and Clinical Neurosciences*, *70*(1), 62–70.	A significant improvement in competence in the management of suicidal students was found. These improvements continued for a month. About 95% of the participants (n = 76) rated the program as useful or very useful and one-third of the participants had one or more chances to utilize their skills within a month.	There was no control arm in the study. The participants were individually selected, which allows for some selection bias. The long-term effectiveness of the study was not tested. The study was carried out in one institution with administrative staff only so the results cannot be generalized. The effects of the interventions on students were not evaluated. The participants already had relatively positive views before the training. The participants ratings were on a single scale and not validated, therefore, making further inferences might not be appropriate.	The study accurately tested the study aims.	The improvements in confidence and management were measured as a single-scale self-reported outcome and thus could not be validated. The results were all assessed using the appropriate statistical measures and the study itself was ethically sound. The self-reported nature of the results decreased the validity and reliability.	There were significant improvements in competence and confidence in managing suicidal students, as well as improvements in the behavioural intention of the teachers for a month following the training. This would suggest the usefulness of the program for improving participants’ attitudes.	Training programs shown to be effective even in areas where there is a positive view on MHP that students may face. The research should focus on student outcomes in the future.
[29]	Hashimoto, N., Takeda, H., Fujii, Y., Suzuki, Y., Kato, T. A., Fujisawa, D., … & Kusumi, I. (2021). Effectiveness of suicide prevention gatekeeper training for university teachers in Japan. *Asian journal of psychiatry*, *60*, 102661.	Eighty-one (81) university teachers were trained, 63 had a 1 h mental health lecture and 18 received the authors’ gatekeeper training. The Suicide Intervention Response Inventory (SIRI) was used for measuring the management of students with suicidal thoughts. The two groups were compared. The participants who received the gatekeeper training were more confident and had better skills.	No follow-up measurement was carried out and the authors could not determine how long the effects lasted. Also, the authors did not assess behavioural change. The training could have been longer in order to have greater impact on behavioural change.	The methodology was quantitative and compared two groups who had received different types of training. It was not clear if this was a randomised control trial as the groups were not randomly selected and the participants did not complete questionnaires before and after but only after the trainings.	The results were statistically analysed and presented in detail in a table, showing statistical significance.	Results showed that focused training can help university teachers gain more confidence and competence regarding recognising the students who are at risk of suicide. This is important provided that mental illness and suicidal ideation are high among university students.	The study filled in an identified gap in the literature because the authors’ previous study was conducted with staff, and they aimed to explore the situation with university teachers.
[30]	Zinzow, H. M., Thompson, M. P., Fulmer, C. B., Goree, J., & Evinger, L. (2020). Evaluation of a brief suicide prevention training program for college campuses. *Archives of suicide research*, *24*(1), 82–95.	Students exhibited a greater increase in gatekeeper behaviour, in comparison to non-students. Large changes were observed on publicizing suicide prevention information and having informal conversations about suicide with students, and 76% had engaged in gatekeeper behaviour at follow-up. Declines on knowledge and self-efficacy from post-test to follow-up highlight the importance of booster sessions and complementary programming.	There is a lack of comparison groups hindering the ability to draw causal conclusions in regard to the impact of the training on the study outcomes. A larger scale of participants is needed (faculty and staff) to fix the discrepancies between the training groups. Most of the student participants were resident assistants (RAs). This may limit the ability to generalize to broader student populations as RAs hold a greater responsibility and may apply the practice skills at a greater frequency. There was a lack of demographic information (race, age, gender) that hinders the ability to determine if the training’s effectiveness varies between the different demographics. The knowledge and self-efficacy measure appeared to be assessing a large number of factors with a small number of items.	The methods accurately explored the aims of the study.	The study followed proper ethical protocols. They incorporated suitable statistical tests (repeated measure ANOVAS) to calculate their data, the calculations to the datasets were also provided. The data and findings were valid and had high reliability.	Findings offer support for the potential efficacy of a brief prevention program, with promising effects on several suicide prevention behaviours.	Widespread participation from students and employees on college campuses could be the next step in suicide prevention training and prevention strategies.
